# Differential effects of rat ADSCs encapsulation in fibrin matrix and combination delivery of BDNF and Gold nanoparticles on peripheral nerve regeneration

**DOI:** 10.1186/s12868-021-00655-y

**Published:** 2021-08-12

**Authors:** Shahnaz Razavi, Maliheh Jahromi, Elham Vatankhah, Reihaneh Seyedebrahimi

**Affiliations:** 1 Department of Anatomical Sciences, School of Medicine, Isfahan University of Medical Sciences, Isfahan, Iran; 2 Department of Biological Systems, Faculty of New Technologies Engineering, Zirab Campus, Shahid Beheshti University, Tehran, Iran

**Keywords:** Adipose derived stem cells, Fibrin matrix, Gold nanoparticle*s*, Sciatic nerve regeneration, Neurotrophic factors

## Abstract

**Background:**

Fibrin as an extracellular matrix feature like biocompatibility, creates a favorable environment for proliferation and migration of cells and it can act as a reservoir for storage and release of growth factors in tissue engineering.

**Methods:**

In this study, the inner surface of electrospun poly (lactic-co-glycolic acid) (PLGA) nanofibrous conduit was biofunctionalized with laminin containing brain derived neurotrophic factor (BDNF) and gold nanoparticles in chitosan nanoparticle. The rats were randomly divided into five groups, including autograft group as the positive control, PLGA conduit coated by laminin and filled with DMEM/F12, PLGA conduit coated by laminin and filled with rat-adipose derived stem cells (r-ADSCs), PLGA conduit coated by laminin containing gold-chitosan nanoparticles (AuNPs-CNPs), BDNF-chitosan nanoparticles (BDNF-CNPs) and filled with r-ADSCs or filled with r-ADSCs suspended in fibrin matrix, and they were implanted into a 10 mm rat sciatic nerve gap. Eventually, axonal regeneration and functional recovery were assessed after 12 weeks.

**Results:**

After 3 months post-surgery period, the results showed that in the PLGA conduit filled with r-ADSCs without fibrin matrix group, positive effects were obtained as compared to other implanted groups by increasing the sciatic functional index significantly (*p* < 0.05). In addition, the diameter nerve fibers had a significant difference mean in the PLGA conduit coated by laminin and conduit filled with r-ADSCs in fibrin matrix groups relative to the autograft group (*p* < 0.001). However, G-ratio and amplitude (AMP) results showed that fibrin matrix might have beneficial effects on nerve regeneration but, immunohistochemistry and real-time RT-PCR outcomes indicated that the implanted conduit which filled with r-ADSCs, with or without BDNF-CNPs and AuNPs-CNPs had significantly higher expression of S100 and MBP markers than other conduit implanted groups (*p* < 0.05).

**Conclusions:**

It seems, in this study differential effects of fibrin matrix, could be interfered it with other factors thereby and further studies are required to determine the distinctive effects of fibrin matrix combination with other exogenous factors in peripheral nerve regeneration.

## Background

A nerve guidance conduit is an alternative to autologous nerve grafting for peripheral nerve regeneration [1]. Among the many different materials used to make the nerve conduit, poly (lactic-co-glycolic) acid (PLGA) is a composite polyester approved by the Food and Drug Administration (FDA) [2].

Furthermore, PLGA is a hydrophobic polymer and has disadvantages, including lack of enough adhesion sites for cell binding and growth of neurites [3]. Therefore, it is important to improve the hydrophilic property of PLGA scaffold surfaces and modify them with an extracellular matrix like laminin [4, 5]. Laminin can be used as a natural material in the inner surface of PLGA nerve conduit to stimulate neurite outgrowth [6], it can promote cell adhesion and proliferation rate on the PLGA sheet [7].

Fibrin matrix has been used as a filler within the lumen of conduits with biodegradability, biocompatibility, non-reactiveness, lower toxicity and stability properties which may impact the tissue repair in a positive pathway [8]. A study reported that application of autologous fibrin glue is valuable efficacy in repairing of sciatic nerves injury in rabbits [9]. It has been shown that fibrin containing Schwann cells (SCs) has been used as a luminal filler and can increase axonal repair [10]. Additionally, neurotrophins along with SCs suspended in fibrin matrix have been used for rat sciatic nerve regeneration [11]. Moreover, fibrin matrix as a natural polymer may be appropriate for this purpose via retaining the cells in the damage zone, providing interaction of cells with extracellular matrix and physical connection to the nerve end [12].

Adipose-derived stem cells (ADSCs) are the promising candidate to be applied as cellular components in a nerve conduit [13]. Therefore, we used rat adipose-derived stem cells (r-ADSCs) to promote nerve repair, owing to the features of secreting growth factors, myelin formation, and improving nerve repair [14, 15].

It has been shown that factors like brain derived neurotropic factor (BDNF) contribute to formation of neurites, increase axonal outgrowth, prevent apoptosis, and form chemotactic factors guiding regenerating axon after nerve damage [16, 17].

In addition, one previous study showed that gold nanoparticles (AuNPs) could improve axonal growth and myelination, as well as prevent neuronal death [18].

Recently, it has been proven that some factors, such as AuNPs can be added on immature neuronal cell line and stimulate cell adhesion, proliferation, differentiation, stimulate axonal elongation, and sprouting axons [19].

To optimize the transfer of r-ADSCs in nerve conduit, a matrix is need to fill the nerve conduit, leading to homogenous distribution of cells over the conduit [20]. This matrix must have some properties such as preserving the cells, not disturbing the axonal growing pathway into the lumen of the conduit, being consistent with cell proliferation, and remaining alive [21, 22].

In our previous study, we found that coating of BDNF and AuNPs encapsulated in chitosan on nanofibers lead to their continuous release for 7 days and this could enhance proliferation and differentiation of adipose derived stem cells into Schwann cells in vitro [23, 24].

Therefore, we decided to fabricate PLGA nanofibrous scaffold with random outer surface and aligned with inner surface orientation by electrospinning. The electrospun nanofiber films were made into nerve conduits after being coated with laminin containing BDNF and AuNPs encapsulated in chitosan nanoparticles, in the inner surfaces of PLGA nerve conduits. Then, r-ADSCs were loaded in fibrin gel and injected into PLGA conduit implanted into transected sciatic nerve rat model. We assessed effects of encapsulation cells in fibrin matric and combination of gold-chitosan nanoparticles (AuNPs-CNPs) and BDNF-chitosan nanoparticles (BDNF-CNPs) factors on sciatic nerve regeneration at 12-weeks post-surgery.

## Results

### Characterization of PLGA nerve conduit and injected r-ADSCs

**Fig. 1:**
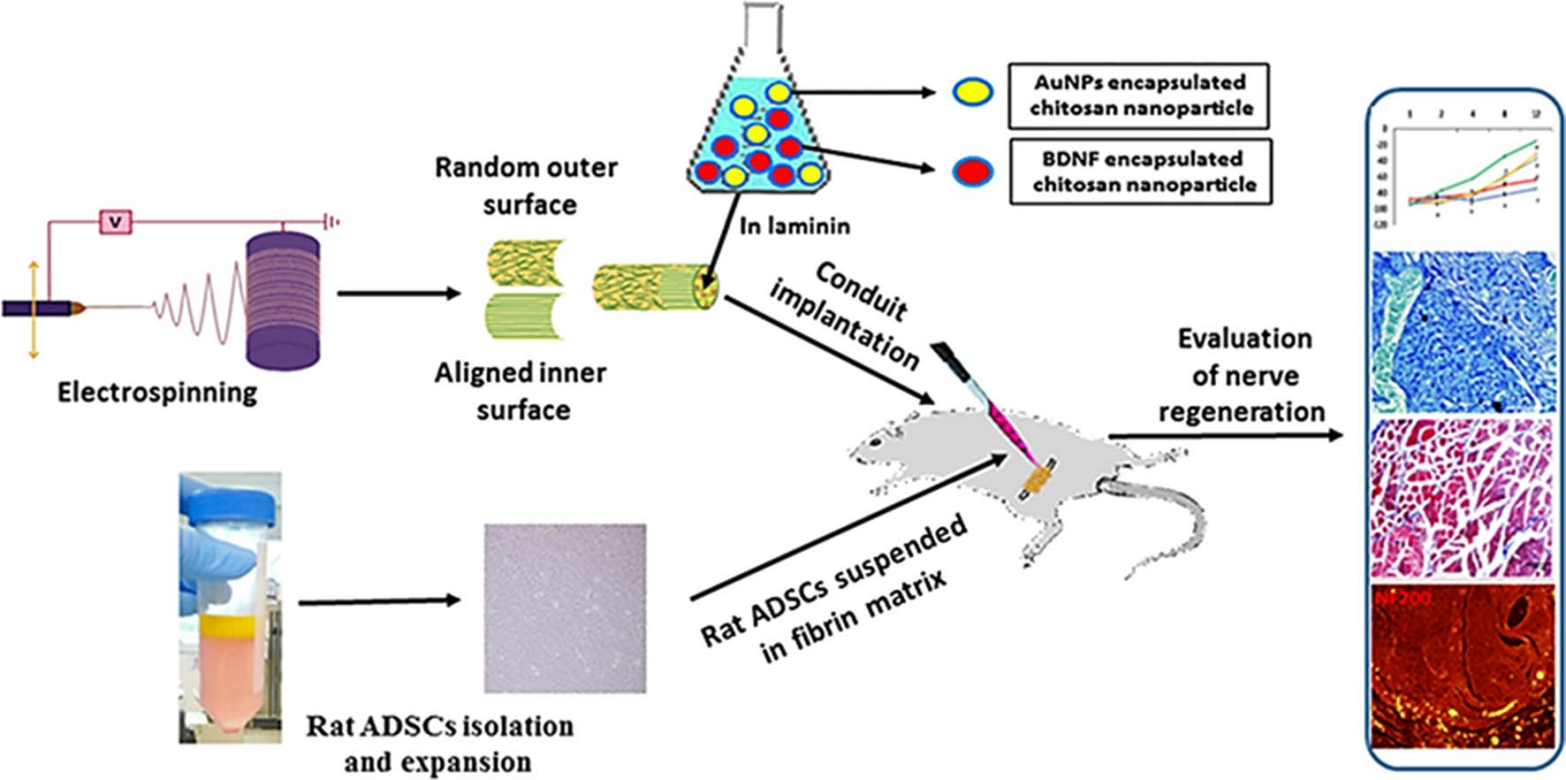
Schematic diagram of PLGA scaffold with aligned inner surface and random outer surface orientated fibers were fabricated by the electrospinning method. The inner surfaces of PLGA nerve conduit coated with laminin containing BDNF and AuNPs encapsulated in chitosan nanoparticles were rolled up to form a tubular nerve conduit. Then, the fibrin gel was loaded with r-ADSCs and injected into the lumen of the PLGA conduit implanted into the 10 mm transected sciatic nerve rat model. The assessment of sciatic nerve regeneration was performed by immunohistochemistry staining, histological and behavioral tests after 3 months post-surgery. The schematic image shown is the authors’ work

The schematic of fabrication of nerve conduit was illustrated in Fig. 1, the inner surfaces of PLGA nerve conduit coated with laminin containing BDNF and AuNPs encapsulated in chitosan nanoparticles were rolled up to form a tubular nerve conduit. Then, fibrin gel was loaded with isolated r-ADSCs and injected into the lumen of the PLGA conduit implanted into the 10 mm transected sciatic nerve rat model. The assessment of sciatic nerve regeneration was performed by immunohistochemistry staining, histological and behavioral tests after 3 months post-surgery.

**Fig. 2:**
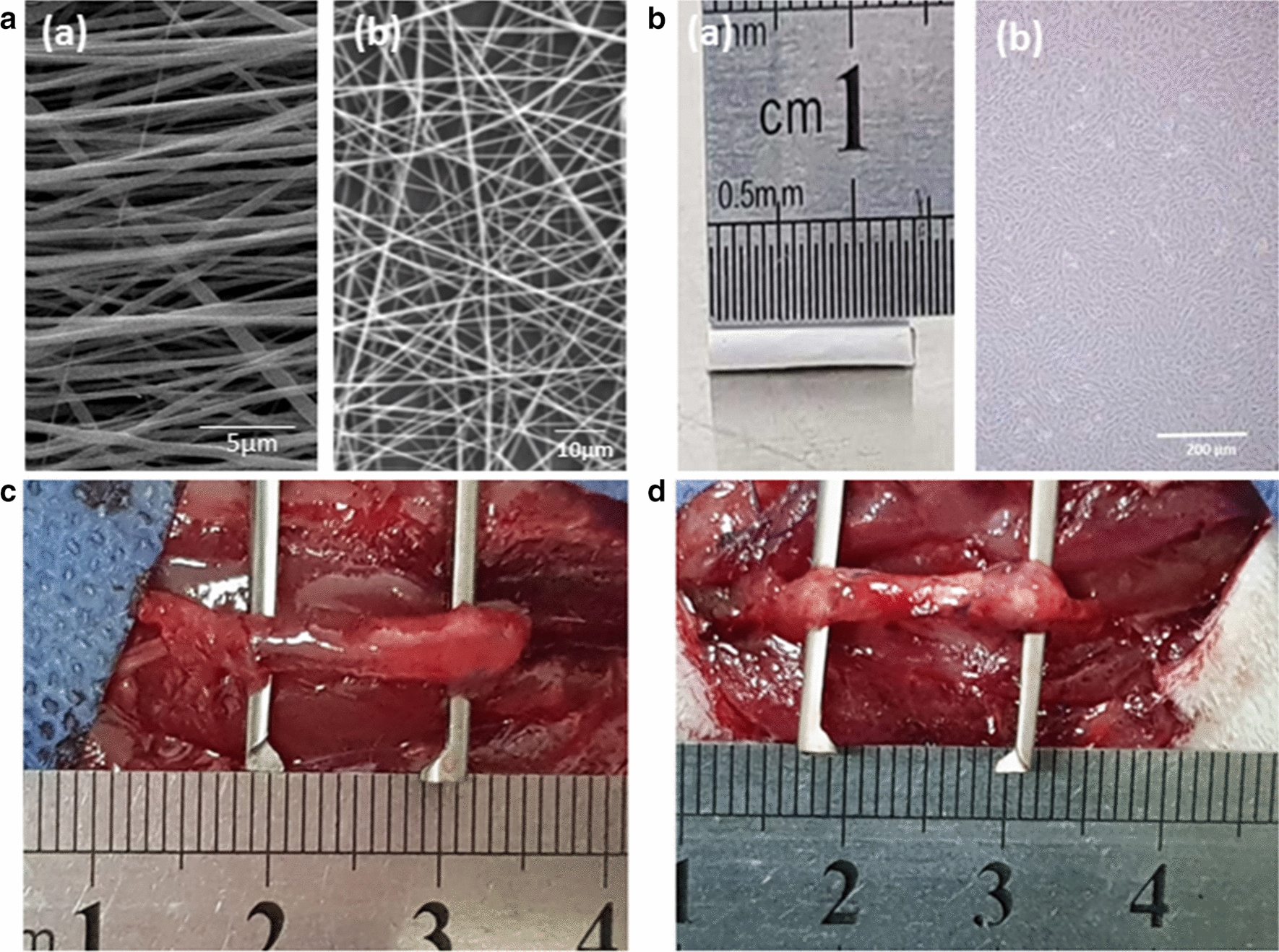
SEM micrographs of electrospun laminin-coated PLGA conduit (**A**); the inner surface of the membrane contained aligned fibers (a) (scale bar is 5 µm); while the outer surface consisted of randomly arranged PLGA fibers (b) (scale bar is 10 µm). The size of nerve conduit (**B**a); After 3 passage primary cultured r-ADSCs were exhibited irregular, flat, and spindle-shape fibroblast-like morphology under light microscopy (**B**b); conduit-implanted in 10 mm rat sciatic nerve gap 12 weeks after surgery in PLGBC (**C**) and PLGBCF (**D**) groups

The structure and morphology of fabricated scaffolds were observed using a scanning electron microscope (SEM)**.** The inner surface of PLGA that directly contacted the growing nerve was electrospun in an aligned form, while the outer surface had randomly oriented nanofibers (Fig. 2A). Furthermore, the SEM image showed that the overall thickness of the PLGA scaffold was approximately ~ 74 µm (using an Image J analysis software). While, the mean thickness of laminin-coated sheets with BDNF/AuNPs encapsulated chitosan nanoparticles was measured ~ 78 µm. Our results indicated that the fabricated conduits had an internal diameter of 2 mm with a length of ~ 1.4 cm (Fig. 2Ba). After 3 passage primary cultured r-ADSCs were exhibited irregular, flat, and spindle-shape fibroblast-like morphology under light microscopy (Fig. 2Bb).

There were no signs of inflammation in the animals and no macroscopic evidence of inflammation at the implanted site. In the PLGBC group, the presence of three factors such as AUNPs, BDNF and r-ADSCs with the conduit exhibited more proper appearance of nerve formation (Fig. 2C), whereas the injection of cells with fibrin (PLGBCF) created an incomplete repair morphology in the nerve (Fig. 2D).

### Sciatic function index outcomes

**Fig. 3:**
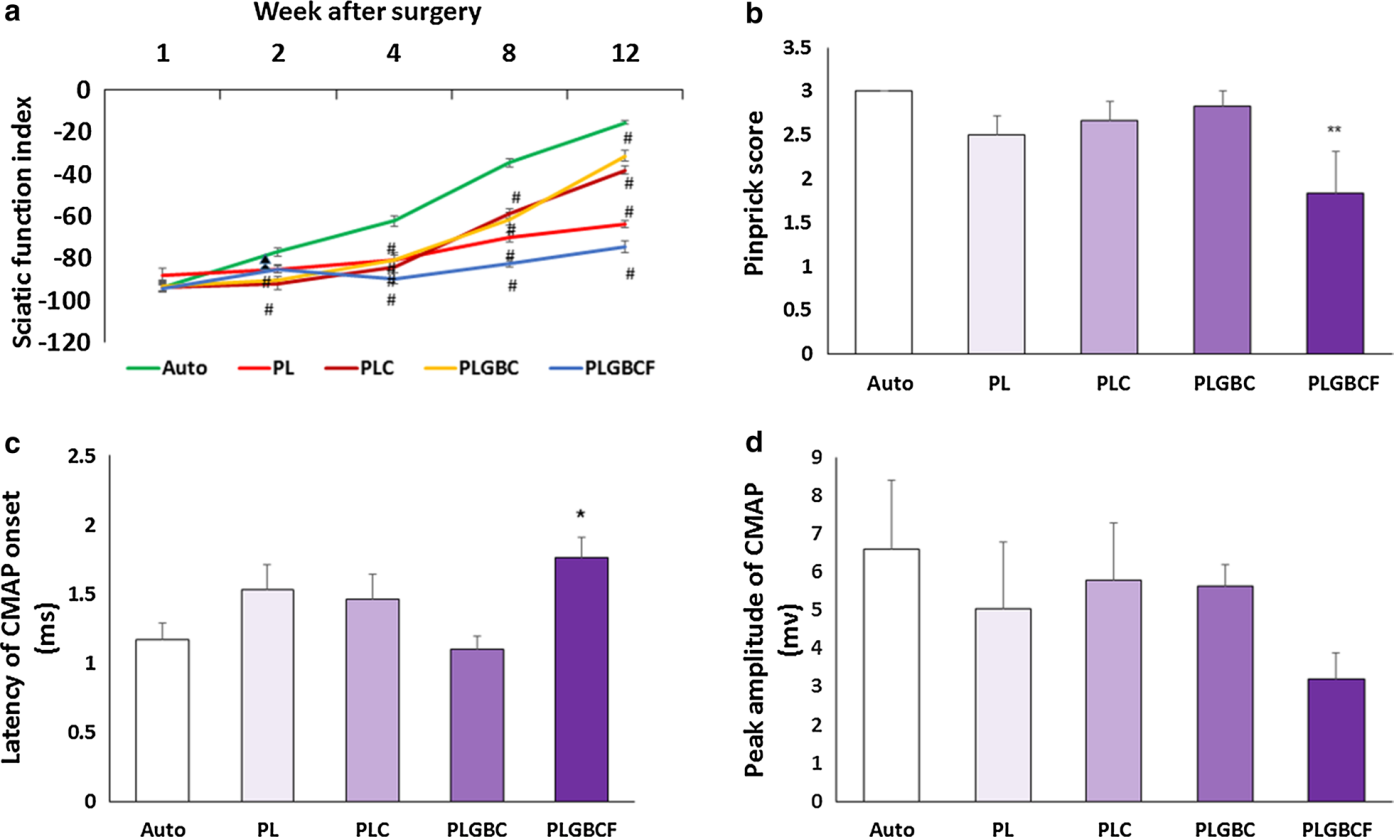
Comparison of the mean of sciatic functional index (SFI) 1, 2, 4, 8, and 12 weeks after surgery (p < 0.001#) (**A**); pinprick score (**B**); the latency (**C**) and CMAP ampilitude index (**D**). Autograft (Auto), P (PLGA), L (Laminin), G (AuNPs), B (BDNF), C (Cell), F(Fibrin), (*p < 0.05,  **p < 0.01 the conduit-implanted group compared to the autograft group)

Behavior analysis such as sciatic functional index and pinprick test was performed to assess the sensory and motor recovery of the sciatic nerve in rats, respectively. Motor recovery of the sciatic nerve was evaluated by the sciatic functional index after the 1st, 2nd, 4th, 8th and 12th weeks after surgery (Fig. 3A). All experimental groups improved over time. The autograft group showed a significantly higher SFI value (− 15.44 ± 0.96) than that of the other implanted nerve conduit groups at the 4th, 8th and 12th weeks after surgery (*p* < 0.001). However, three months after the transplantation, repair with PLGBCF had a lower significant SFI value (− 74.44 ± 2.69) than that of all experimental groups (*p* < 0.001). At the 12th week after surgery, the PLGBC group showed a significantly increase mean SFI value (− 31.3 ± 2.34) than that of the other implanted conduit groups (*p* < 0.05).

### Pinprick test outcomes

To assess of sensory recovery, the pinprick test was performed. The results of pinprick test show that there was no significant difference mean pinprick score between autograft group (3 ± 0.0) and PL (2.5 ± 0.2), PLC (2.6 ± 0.2) and PLGBC (2.8 ± 0.1) groups at 12 weeks' post-surgery. However, there was a significant difference mean of pinprick score in the autograft group (3 ± 0.0) compared with PLGBCF (1.8 ± 0.4) group (*p* < 0.01). All autograft animals received score 3 at 12 weeks, regardless of type of nerve repair (Fig. 3B).

### Outcomes of electrophysiological assessment

Twelve weeks following surgery, the CMAP latency and CMAP amplitude were measured on the implanted side in experimental grafted groups by electrophysiological assessment (Fig. 3C–D). Our results revealed that the mean CMAP latency in the PLGBCF group was significantly higher than that of the other implanted and autograft groups (*p* < 0.05), but no statistically significant differences were found between the autograft group and the other treated groups except PLGBCF (Fig. 3C).

The CMAP amplitude ratio showed no significant difference between autograft and conduit implanted groups. In fact, these CMAP amplitude values were higher in the autograft group (6.60 ± 1.8), then the PL (5.04 ± 1.73), the PLC (5.78 ± 1.5) the PLGBC (5.64 ± 0.5) and the PLGBCF (3.21 ± 0.6) groups. However, these differences in the mean of CMAP amplitude values were not significant between the experimental groups (Fig. 3D).

### Histological analyses of gastrocnemius muscle

**Fig. 4:**
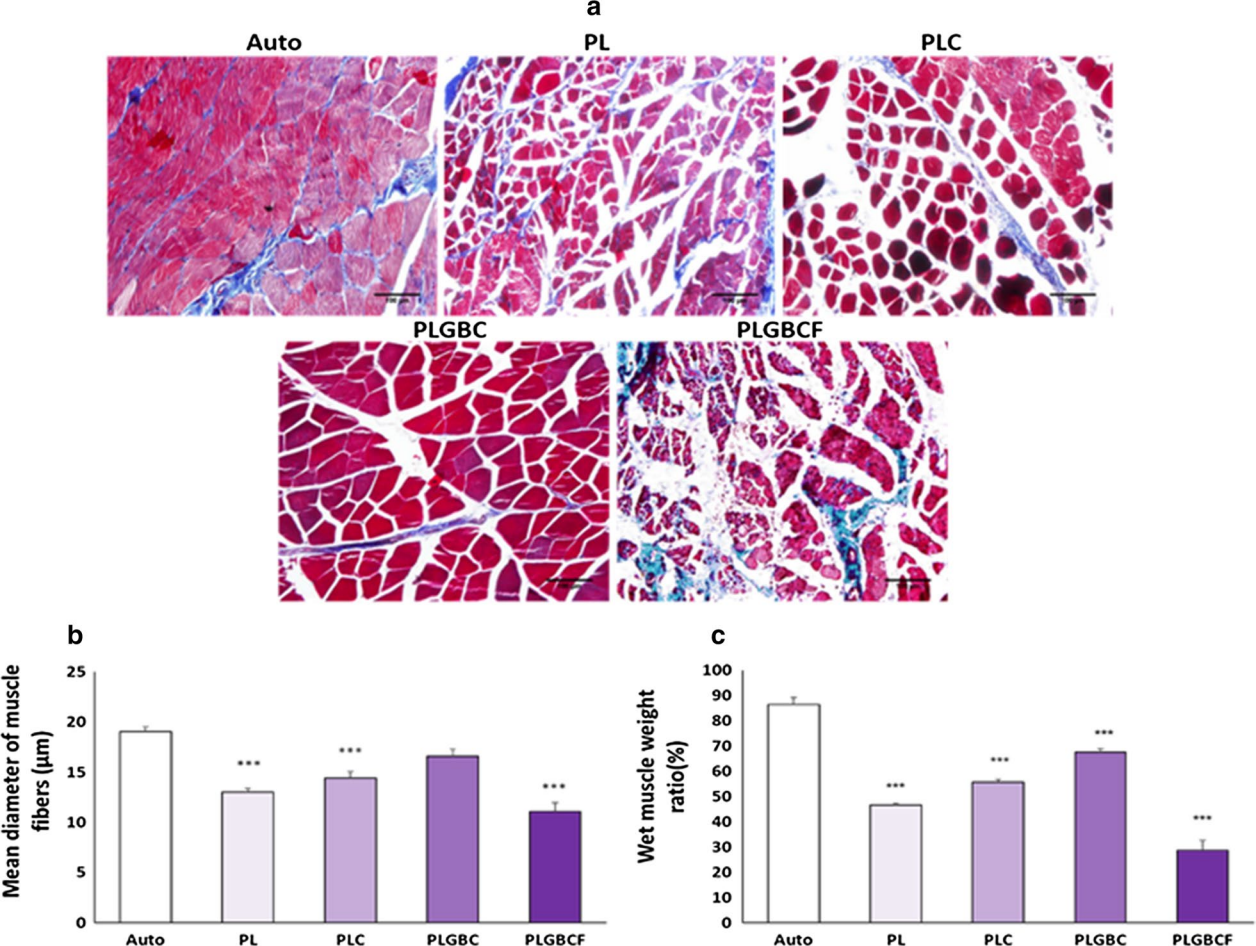
Representative images of transverse sectioned gastrocnemius muscle following Masson's trichrome staining 12 weeks after surgery (scale bars = 100 µm) (**A**); The mean diameters of muscle fibers (**B**); Wet muscle weight ratio (%) (**C**); Autograft (Auto), P (PLGA), L (Laminin), G (AuNPs), B (BDNF), C (Cell), F(Fibrin). (_***_ p < 0.001, the conduit-implanted group compared to the autograft group)

To assess atrophy in the gastrocnemius muscle, owing to sciatic nerve transection, Masson's trichrome staining was performed for muscle in five groups (Fig. 4A). Denervation of target muscles led to decrease muscle fiber diameters. Compared to muscle morphology in the autograft group, the mean muscle fibers diameter suffering from denervation was degenerated in the PLGBCF group (11.06 ± 0.9) and showed a significant smaller muscle cell diameter (*p* < 0.001). However, the mean muscle fibers diameter of PL (13.02 ± 0.3), PLC (14.41 ± 0.6) and PLGBCF groups had a significant decrease compared to that in the autograft group (19 ± 0.4) (*p* < 0.001), but the mean of muscle fiber diameter in PLGBC group (16.5 ± 0.7) was not significant different with the autograft group (19 ± 0.4) (*p* > 0.05) (Fig. 4B).

Twelve weeks after implantation, the mean wet weight of gastrocnemius muscle in the autograft group (86.52 ± 2.6%) was significantly increased than in the conduit-implanted groups (*p* < 0.001). In addition, the mean muscle mass in the PLGBCF group (28.68 ± 3.9%) was significantly smaller than that in the autograft and other experimental groups (*p* < 0.001). The mean weight of the gastrocnemius muscle in the PLGBC group (67.49 ± 1.3%) was significantly higher than that in PL and PLGBCF groups (*p* < 0.001). Furthermore, the atrophy in PLGBC was significantly less than that in the PLC group (*p* < 0.01) (Fig. 4C).

### Nerve histomorphometry

**Fig. 5:**
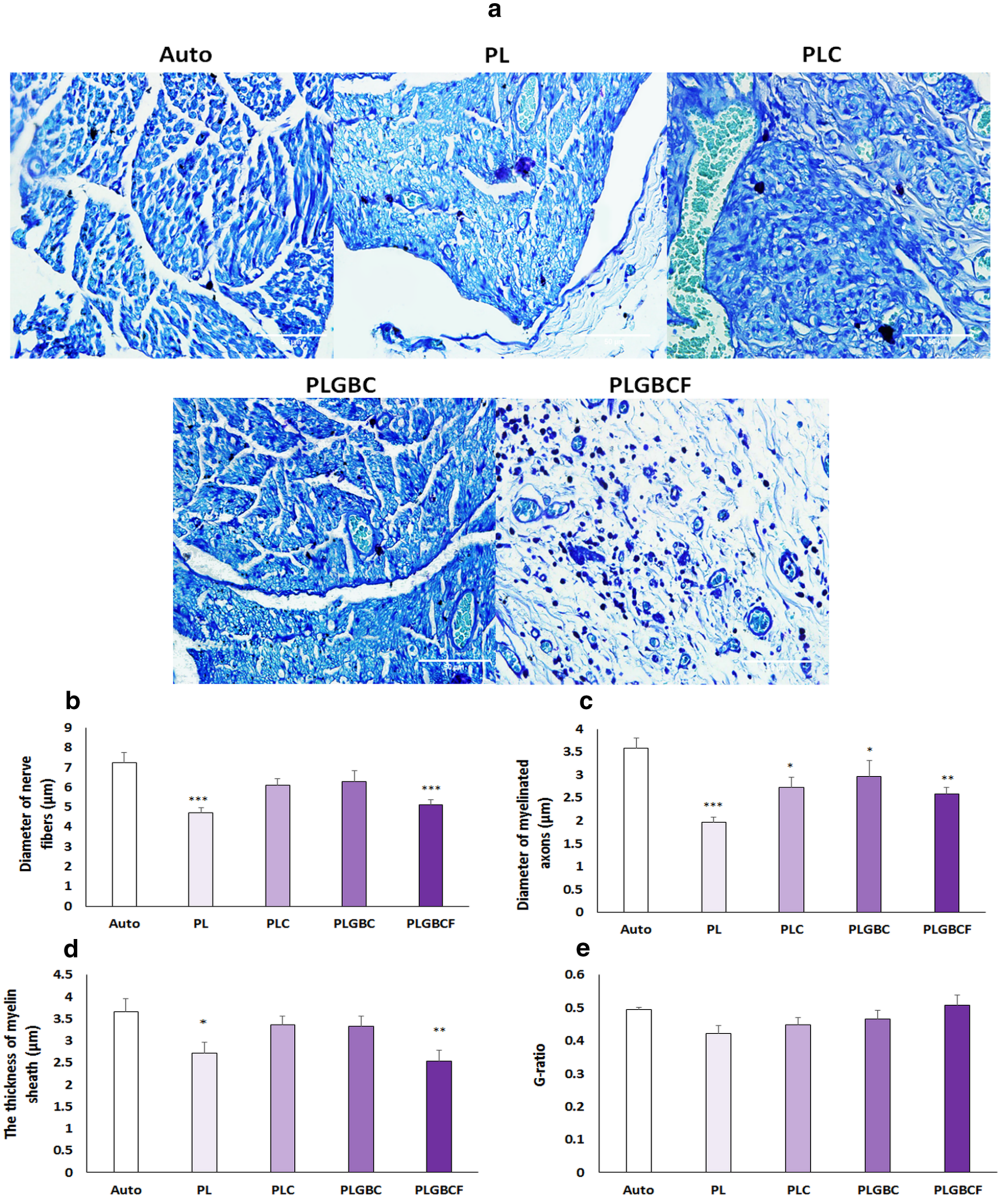
Photomicrograph regenerated nerves using toluidine blue staining 12 weeks after surgery(scale bars = 50 µm) (**A**); and quantitative analysis of the mean nerve fiber diameter (**B**), myelinated axon (**C**), thickness of myelin sheath (**D**) and G-ratio (**E**) in different groups; Autograft (Auto), P (PLGA), L (Laminin), G (AuNPs), B (BDNF), C (Cell), F(Fibrin). (*p < 0.05, **p < 0.01,  ***p < 0.001 the conduit-implanted group compared to the autograft group)

Figure 5A depicts the results of toluidine blue staining for the cross-section of regenerated nerves. Numerous fibers of regenerated nerves can be found in the autograft group. We observe that the mean nerve fibers diameter had no significant difference between PLC (6.08 ± 0.3) and PLGBC (6.2 ± 0.5) groups with the autograft group (7.2 ± 0.5) (*p* > 0.05), but this difference mean was significant between PL and PLGBCF (5.1 ± 0.2) groups with the autograft (7.2 ± 0.5) group (*p* < 0.001) (Fig. 5B). Furthermore, all conduit implanted groups showed that the mean diameter of myelinated axons was significantly smaller than that in autograft (3.5 ± 0.2) groups; this difference was more evident in PL (1.9 ± 0.1) and PLGBCF (2.5 ± 0.1) groups (*p* < 0.001) than PLC (2.7 ± 0.2) and PLGBC (2.9 ± 0.3) groups (*p* < 0.05) with autograft groups (Fig. 5C). Additionally, quantitative analysis shows that the mean thickness of myelin sheet in PL (2.7 ± 0.2) and PLGBC (3.3 ± 0.2) groups was not significantly different from that of the autograft (3.6 ± 0.2) group (*p* > 0.05), but there was a significant different mean in this analysis between PL (*p* < 0.05) and PLGBCF (2.5 ± 0.2) (*p* < 0.01) groups and autograft group (Fig. 5D). Finally, there was no significant different mean in the G-ratio between all conduit-implanted groups and autograft groups (Fig. 5E).

### Immunohistochemical analysis of regenerated nerves

**Fig. 6:**
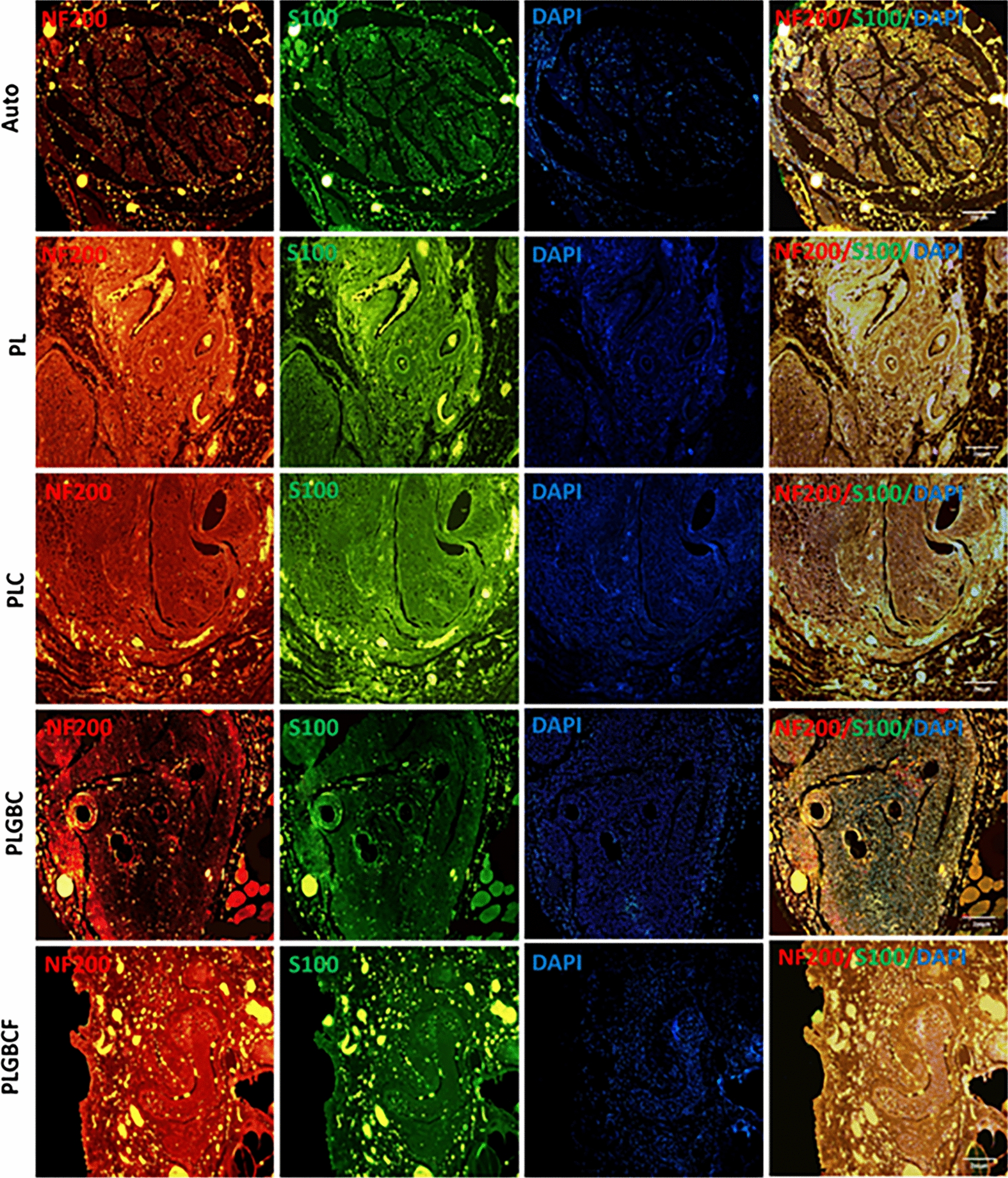
The representative images of regenerated nerves in the conduit; Double-immunohistochemical staining of regenerated nerve transvers sections with NF-200/S-100 in each group; Neurofilaments were labeled with NF-200 exhibiting a red color. Schwann cells were labeled with S-100 and appears as green and cell nuclei were labeled with DAPI (blue) (scale bars = 200 µm) in different groups; Autograft (Auto), P (PLGA), L (Laminin), G (AuNPs), B (BDNF), C (Cell), F(Fibrin)

**Fig. 7:**
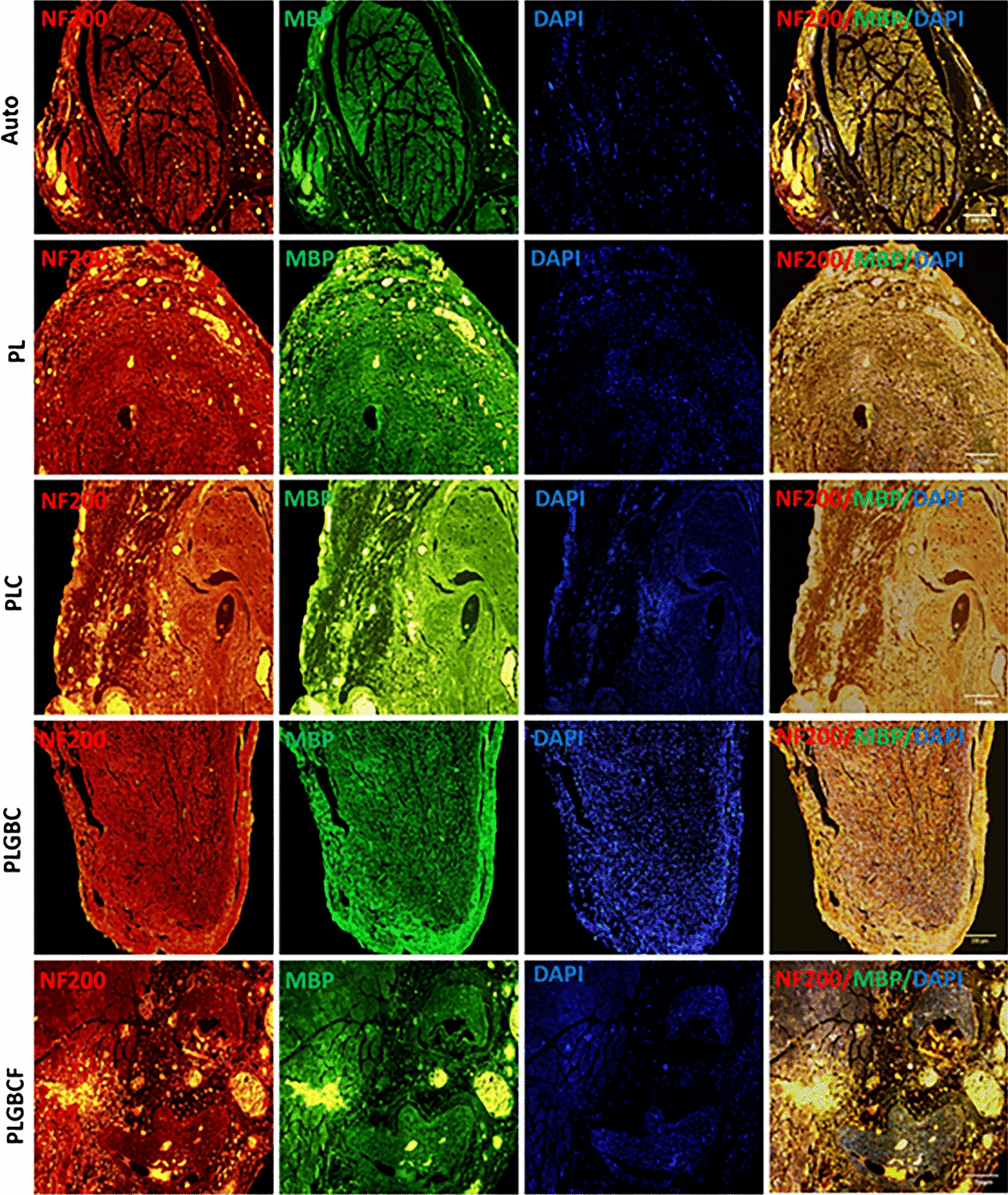
The representative images of regenerated nerves in the conduit; Double-immunohistochemical staining of regenerated nerve transvers sections with NF-200/MBP in each group; Neurofilaments were labeled with NF-200 exhibiting a red color. MBP appear as green and cell nuclei were labeled with DAPI (blue) (scale bars = 200 µm)) in different groups; Autograft (Auto), P (PLGA), L (Laminin), G (AuNPs), B (BDNF), C (Cell), F(Fibrin)

At 12th week, the presence of r-ADSCs throughout the gap area is clearly apparent in longitudinal sections, suggesting that they contributed to the process of tissue regeneration in vivo. The micrographs depict the immunohistochemical cross sections of the middle region of the implanted laminin coated PLGA conduit loaded with r-ADSCs in different experimental groups. Then, to evaluate the immunopositivity expression intensity for S100, MBP and NF200 cells in autograft and all implanted conduits, three immunohistochemical markers, S100, MBP and NF200, were applied to all the samples of experimental groups (Figs. 6 & 7). To visualize axon fibers, neurofilaments were labeled with NF-200 exhibiting a red color. Schwann cells labeled with S100 and cells labeled with MBP appeared as green. Finally, cells nuclei were labeled with DAPI exhibiting a blue color*.* Microscopic fields were randomly selected from each slice for measuring the immunohistochemical intensity value of positive cells.

The comparison of the mean percentage of intensity value S100, MBP and NF200 intensity in experimental groups relative to autograft group have shown that in Table 1.

**Table 1:** Comparison of the mean percentage of intensity value for MBP, NF200, and S100 in different conduit implanted groups

Groups	Intensity of MBP mean ± SE	Intensity of S100 mean ± SE	Intensity of NF200 mean ± SE
Auto	100	100	100
PL	78.6 ± 0.88***	89.18 ± 0.43***	78.22 ± 0.51***
PLC	96.47 ± 0.48**	102.20 ± 0.61	96.51 ± 0.86*
PLGBC	99.29 ± 0.43	106.25 ± 0.63 ***	125.17 ± 1.16***
PLGBCF	45.05 ± 0.52***	48.07 ± 0.58***	76.18 ± 0.60***

Autograft (Auto), P (PLGA), L (Laminin), G (AuNPs), B (BDNF), C (Cell), F(Fibrin)

(*p < 0.05, **p < 0.01, ***p < 0.001. The mean percentage of intensity value of positive cells in conduit-implanted groups compared to autograft group)

We found that the mean intensity of S100 positive cells was significantly higher in the PLGBC group (106.25 ± 0.63) than in PL (89.18 ± 0.43), PLGBCF (48.07 ± 0.58**)** groups and the autograft group (*p* < 0.001) 12 weeks after nerve after grafting. While, the intensity of S100 positive cells in the PLGBCF group is less than that in the other treated groups (48.07 ± 0.58).

The statistics of MBP-positive intensity indicated a significant difference between PL (78.6 ± 0.88) and PLGBCF (45.05 ± 0.52) groups and other experimental groups (*p* < 0.001), whereas this intensity in the PLC group (96.47 ± 0.48) was significantly different from that in the autograft group (*p* < 0.01). Furthermore, in PLGBC groups, it was not significantly different from that of the autograft group (*p* > 0.05) (Table 1). While the mean percentage of intensity of NF200 positive cells in PLGBC was higher than that in the other experimental groups (125.17 ± 1.16), and this difference was significant (*p* < 0.001). Meanwhile, a significant difference mean of the NF200 intensity was obtained in PL, PLC and PLGBCF groups compared with the autograft groups (*p* < 0.05) (Table 1)**.**

### Quantitative real-time RT- PCR

**Fig. 8:**
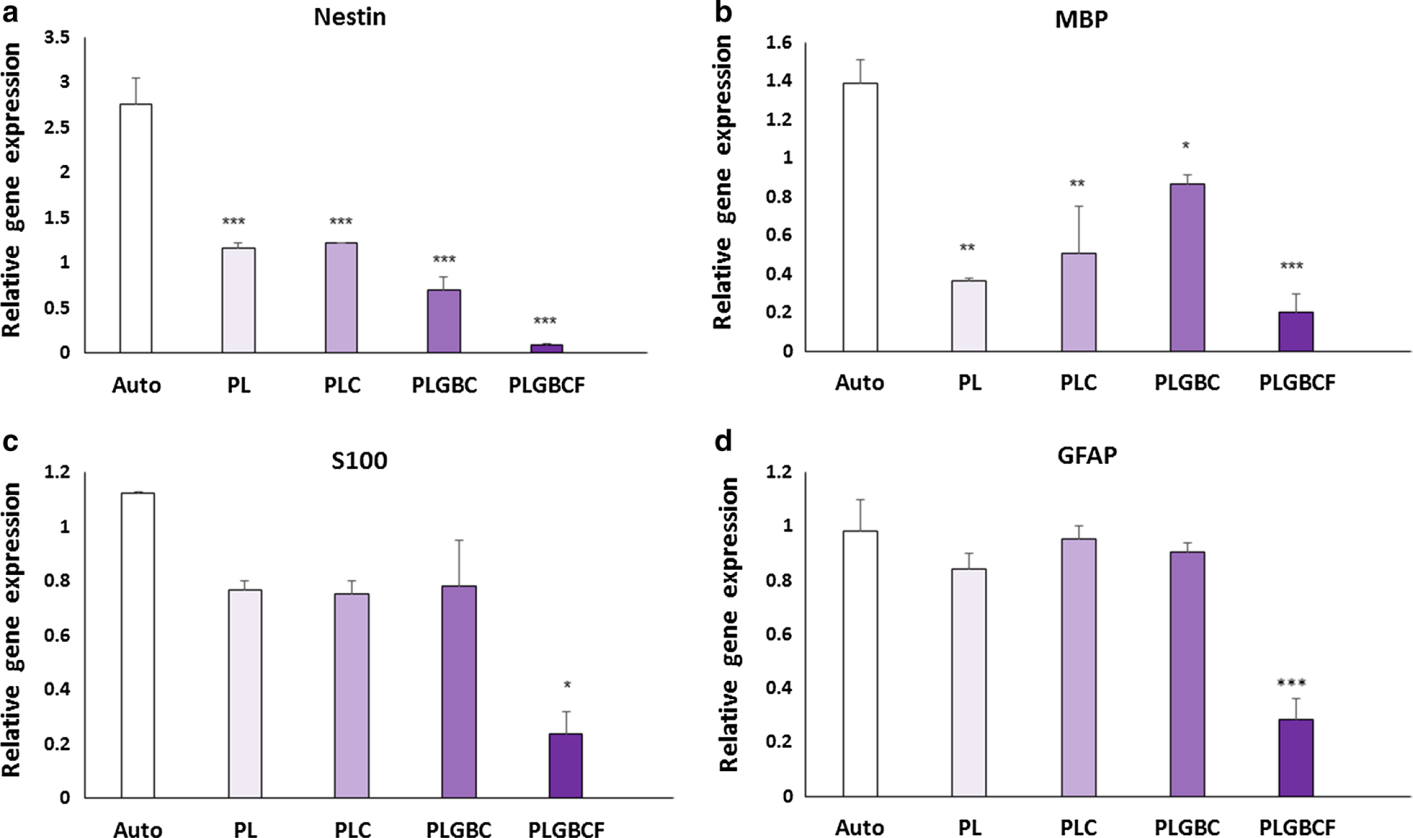
Comparative analysis mean of the level of Nestin, MBP, S100, and GFAP expression in different groups evaluated by real-time RT-PCR technique (**A**–**D**); Autograft (Auto), P (PLGA), L (Laminin), G (AuNPs), B (BDNF), C (Cell), F(Fibrin). (_*_p < 0.05, _**_p < 0.01, _***_p < 0.001, the conduit-implanted group compared to the autograft group)

Twelve weeks after surgery, the level of neural precursor (Nestin), glial cell (GFAP), specific Schwann cell (S100β), and myelinization ability (MBP) mRNA was measured in all experimental groups by the real-time PCR (Fig. 8A–D). The Nestin in PL (1.17 ± 0.05), PLC (1.2 ± 0.005), PLGBC (0.7 ± 0.15) and PLGBCF (0.09 ± 0.01) groups and MBP mRNA levels in PL (0.3 ± 0.01), PLC (0.5 ± 0.2), PLGBC (0.8 ± 0.04) and PLGBCF (0.2 ± 0.09) expression was significantly downregulated as compared to that in the autograft groups in Nestin (2.7 ± 0.2) and MBP (1.3 ± 0.1) expression (*p* < 0.05) (Fig. 8A & B), while, for S100β in PL (0.7 ± 0.03), PLC (0.7 ± 0.04), PLGBC (0.7 ± 0.1) and PLGBCF (0.2 ± 0.08) groups and GFAP in PL (0.8 ± 0.06), PLC (0.9 ± 0.04), PLGBC (0.9 ± 0.03) and PLGBCF (0.2 ± 0.07) groups, there was no statistically significant difference mean between the conduit-implanted groups and the autograft groups in S100β (1.1 ± 0.005) and GFAP (0.9 ± 0.1) expression (*p* > 0.05) except in the PLGBCF group (*p* < 0.05) (Fig. 8C & D).

## Discussion

In the present study, the PLGA scaffold with aligned inner surface and random outer surface orientated fibers were fabricated by the electrospinning method. The inner surfaces of PLGA nerve conduit coated with laminin containing BDNF and AuNPs encapsulated in chitosan nanoparticles were rolled up to form a tubular nerve conduit. Then, the fibrin gel was loaded with r-ADSCs and injected into the lumen of the PLGA conduit implanted into the 10 mm transected sciatic nerve rat model. The nerve repair status with or without of r-ADSCs, fibrin matrix and BDNF/AuNPs was investigated by different methods in a 12-week post-surgery period.

In the previous study, we demonstrated that the releasing rate of BDNF was 83.28 ± 2.22 (%) of chitosan/ tripolyphosphate (TPP) particles during the 7 days [24]. Also, a sustained release of BDNF and AuNPs from PLGA scaffolds functionalized with coated laminin containing chitosan nanoparticles encapsulating either AuNPs or BDNF was detected over a period of 7 days. The release rate of BDNF and AuNPs from nanofibers was 74 ± 2.42% and 47.24 ± 1.78%, respectively. In addition, our results showed that biofunctionalized scaffold enhanced proliferation, differentiation and myelinization of h-ADSCs into SC-like cells [23].

Previously research has demonstrated that, BDNF may be effect on ADSCs differentiation into Schwann-like cells phenotype during 7 days and growth factors released by differentiated SCs help to neurite outgrowth [25].

Therefore, our hypothesis was that it might be effective in peripheral nerve regeneration.

Strategies of using the PLGA conduit containing with ADSCs were useful to regenerate transected nerve. It is shown that, the ADSCs results increase growth factor secretion, axonal myelination and finally nerve repair [14, 15]. Furthermore, exogenous factors such as AuNPs and BDNF were used for more recovery in nerve repairing [16, 18].

Fibrin matrix is one of these hydrogels, which has been extensively used for nerve regeneration, and it has been shown that the nerve conduits fabricated by fibrin affect axonal growth and myelination [26, 27].

According to one study, autologous fibrin glue in combination with several growth factors might effectively increase peripheral nerve regeneration in 15 mm rabbit peroneal nerve defect [9].

In addition, autologous fibrin glues have some advantages, including no risk of infection or hypersensitivity. Since fibrin glues are usually prepared from whole blood therefore, their use leads to increased risk of transmission of the pathogen from the donor to the recipient [28]. However, disadvantages of available commercial fibrin glues include viral transmission risk, expensiveness, reduced bioactivity and growth factors, which are not present in commercial products [29, 30].

Rafijah et al. have demonstrated that the use of collagenous conduits filled with fibrin glue in 10 mm rat sciatic nerve gap promotes axonal repair and functional recovery 12 weeks after surgery [31]. Therefore, according to the previous studies, we purposed that laminin coated PLGA nerve conduit containing BDNF and gold nanoparticle was filled with r-ADSCs in fibrin matrix, can improve peripheral nerve regeneration.

We observed that the PLGA conduit was degraded completely 12 weeks after implantation.

The evaluation of electrophysiological in all implanted groups, as indicator of conduction function of peripheral nerves, was performed for detectable CAMP latency and amplitude analysis. The latency of CMAP represents the thickness of the myelin sheath, whereas the CMAP amplitude shows the number of nerve fibers [32]. The comparison in the CMAP latency between all groups showed that the speed of transport in action potential in PLGBCF group was significantly lower than that of the other groups (*p* < 0.05), but the comparison in the CMAP amplitude between four conduit implanted groups provided further evidence that their functional recovery was closer to that in the autograft group. The lack of amplitude differences mean indicates that the number of regenerated nerve fibers in all groups is approximately similar. Also, the results of SFI and pinprick tests which indicates motor and sensory nerve recovery are consistent with electrophysiologic outcomes.

These documents indicate that the presence of r-ADSCs with BDNF and AuNPs can lead to nerve repair, but the loaded of fibrin along with these factors may have non synergistic effects. Our results are inconsistent with the data obtained by Masgutov et al. indicating that adipose-derived mesenchymal stem cells loaded in fibrin glue promote peripheral nerve regeneration in sciatic nerve injury [12]. In our study, a combination of factors was used that each of them alone increased nerve regeneration, but we have shown that fibrin matrix may interact with other factors and reduce the effect of each other.

Histological and wet weight analyses in gastrocnemius muscle evaluations indicated that PLGBC, PLC, PL and PLGBCF achieved more reconstruction, respectively. However, the results of these methods between conduit-implanted groups were not comparable to those in the autograft group (*p* < 0.001). Nevertheless, the mean diameter muscle fiber in the PLGBC group was not significant in the autograft group (*p* > 0.05). These results are in line with previous researches. McGrath et al. showed that gastrocnemius muscle weight was decreased in the conduit filled with fibrin matrix, but treatment with cyclosporine A or cyclosporine A with human mesenchymal stem cells induced recovery of the muscle weight [33].

In the present study, the nerve histological data showed that the mean thickness of myelin sheath and diameter of nerve fibers were not significantly different mean in PLGBC and PLC groups, compared to in the autograft group (*p* > 0.05). However, the mean diameter of myelinated axons in PLGBC (2.97 ± 0.34) and PLC (2.73 ± 0.22) was higher among the conduit implanted groups but comparisons have different significant with autograft group (*p* < 0.05).

In this study, application of fibrin matrix in conduit hasn't promoted effect in nerve regeneration and despite poor results that indicates the fibrin group is limited nerve regenerated, but, the non-significant difference between all groups in the parameters of G-ratio and amplitude (AMP) was observed. It maybe indicates fibrin can repair sciatic nerve in terms of number of axons and the thickness of the myelin in received conduit.

The mean number of myelinated axons in PL and fibrin groups was significantly lower than that in the autograft group, respectively (*p* < 0.001). However, there were no differences between PLGBC and PLC groups. As a result, in the groups containing AuNPs and BDNF, there were a higher number of axons, suggesting that the encapsulated nanoparticles, due to enhance rate of differentiation of r-ADSCs into SCs, can lead to repair of the damaged nerve.

However, increases in toxicity levels for the use of intraluminal fillers such as fibrin must be kept in mind. Wood et al. used a fibrin matrix which degraded after a four-week period and despite its approximately short residence time showed beneficial effects on nerve repair [34]. This further complements the idea that intraluminal fillers like fibrin are beneficial due to provide a proper bed for supporting of ADSCs function, but only at the early stages of regrowth, after which it can be hypothesized that may be interact with other factors [35], results loss of this effect.

The presence of r-ADSCs in the PLGA conduit in combination with BDNF/AuNPs without the presence of fibrin may lead to their differentiation into SCs and, as a result, high expression of S100 in immunohistochemical and real-time PCR methods.

The results of the expression of MBP and NF200 were confirmed by the rate of S100 expression. Therefore, myelinated axons (MBP positive cells) and regenerating axons (NF200 positive cells) in conduit-implanted groups containing cell and growth factor were higher than those in the other conduit implanted groups, and it seems that in the PLGBCF group, fibrin interfered with other factors, thereby preventing to improve nerve regeneration.

## Conclusion

Overall, we found that laminin-coated PLGA conduit could improve axonal regeneration in peripheral nerve using some exogenous factors such as BDNF, AuNPs, and ADSCs in fibrin matrix. Although there was some evidence that fibrin matrix promoted peripheral nerve regeneration, but distinctive effect of fibrin was not observed in this study. It should be noted that fibrin matrix may interfere with other factors [35]. It is recommended that future works to determine interactions and definitive effect of fibrin matrix along other factors in peripheral nerve regeneration following injury.

## Materials and methods

### Fabrication and characterization of electrospun PLGA nanofibers

All of materials which used in this study were prepared from Sigma-Aldrich, St. Louis, MO, USA except it was mentioned in the text. Electrospun scaffolds with mild modification were fabricated as described in our previous study [23, 36]. PLGA (80:20, Mw: 50,000–75,000) was dissolved in chloroform: N, N-dimethyl formamide (DMF) (Merck, Darmstadt, Germany) mixture with a volume ratio of 80: 20 to obtain a concentration of 20% (w/v). High voltage electric field of 21 kV was used to draw the polymer solution fed at a rate of 250 µL/h into nanofibers over a distance of 15 cm from the needle tip to an aluminum-wrapped rotating drum. The drum rotation speed was gently decreased from 2500 to 300 rpm. Therefore, a highly aligned nanofibrous inner surface was formed (2500 rpm drum rotation), and as the drum rotation speed decreased (300 rpm drum rotation), the fibers randomly orientation on the outer surface were formed.

For microscopic examination of electrospun PLGA fibers by SEM images, electrospun samples were coated with a thin layer of gold and then observed by SEM microscope (SEM, Seron Technology AIS 2500, India).

### PLGA sheets coating with laminin containing BDNF and AuNPs encapsulated chitosan nanoparticles

AuNP (US Research Nanomaterials, Inc, Houston, TX, USA) or BDNF (R&D systems, Minneapolis, MN, USA) encapsulated chitosan (low molecular weight with a deacetylation degree of > 75%) nanoparticles were produced as described in our previous study [36]. 50 ppm AuNPs or 5 µg/ ml BDNF with 0.1% chitosan solution, the solution was mixed on a magnetic stirrer for 15 min. Then, 0.03% tripolyphosphate (TPP) of an aqueous solution as a cross linker was added dropwise into the previous solution.The 20 µg/ ml of laminin was used for supply suspension of chitosan nanospheres containing BDNF or AuNPs, and finally was coated on the PLGA scaffold at 4 °C temperature for 24 h [24].

### Design and fabrication of nerve conduit

PLGA sheet was cut to 14 × 20 mm. The mats were put under a UV lamp for 2 h and coated with chitosan nanospheres encapsulated BDNF or AuNPs mixed by 20 µg/ ml laminin. Next, the electrospun mat rolling 2.5 rounds around a mandrel to create a tubular structure. The edge of conduit was sealed with cyanoacrylate glue. All these procedures were performed under aseptic conditions [36].

### Rat-ADSCs isolation*,* expansion

All of the experimental procedures involving animals were conducted in accordance with the guidelines given by National Institute of Health Guide for the Care and Use of Laboratory Animals, and approved by the Animal Ethics Committee of Isfahan University of Medical Sciences (No: IR.MUI.REC.1396.3.207). Under sterile conditions, the isolated tissue was placed on ice to the cell culture laboratory. Briefly, the adipose tissue surrounding the inguinal region was cut into tiny segments and treated with 0.075% collagenase type I and shaken at 37 °C for 35 to 40 min. The resultant to neutralizing enzyme activity was added to the Dulbecco’s Modified Eagles Medium (DMEM) /F12 (Gibco Grand Island, NY, USA) containing 10% fetal bovine serum (FBS) (Gibco Grand Island, NY, USA) into each tube. After centrifugation (1200 rpm for 5 min), the upper fat tissue layer and supernatant were discarded. The cell pellet was transferred into culture flasks containing DMEM + 10% FBS and transferred into an incubator at 5% CO2 and 37 °C and saturated humidity. After 3–4 days, the medium was refreshed, and the cells were sub-cultured until passage 4. In this study, r-ADSCs obtained from passage 3–4 were used for the experiments.

### Operation procedure and experimental groups

In this study, 40 mature (12-week-old) *Wistar* male rats (weighing ~ 200–250 g) purchased from Pasteur Institute, Tehran, Iran and were housed under standard conditions (at 18–24 °C, 12 h light/dark) with free access to laboratory pellet chow and water. Acclimatization of animals began 10 days before the experiment date. Each rat was kept in one cage during experimental period.

The rats were randomly divided into five groups (each, n = 8), including autograft group (Auto) as positive control, PLGA conduit nanofibrous coated by laminin and filled with DMEM/F12 (PL), PLGA conduit coated by laminin and filled with 2 × 10^6^ r-ADSCs according to previous study [37] (PLC), PLGA conduit coated by laminin containing BDNF-CNPs, AuNPs-CNPs and filled with 2 × 10^6^ r-ADSCs (PLGBC) and PLGA conduit coated with laminin containing BDNF-CNPs, AuNPs-CNPs and inner volume of the conduit filled with 2 × 10^6^ r-ADSCs suspended in fibrin matrix (PLGBCF) transplanted into 10 mm sciatic gap.

Each rat was anesthetized with intraperitoneal injection of a mixture of Xylazine 10 mg/kg and Ketamine 100 mg/kg. The thigh areas on left sides were shaved and sterilized with betadine. Then, the sciatic nerve was exposed by skin and muscle-splitting incision.

The 10 mm length segment of sciatic nerve was removed and PLGA conduit coated by laminin and filled with DMEM/F12 (PL), PLGA conduit coated by laminin and filled with 2 × 10^6^ r-ADSCs for each animal (PLC) and PLGA conduit coated by laminin containing BDNF-CNPs, AuNPs-CNPs and filled with 2 × 10^6^ r-ADSCs for each animal (PLGBC) implanted in different groups.

Also, the 10 mm segment of sciatic nerve was excised, reversed, and without rotation reattached using 10-0 nylon as an autograft group. Finally, in the fibrin matrix group include PLGA conduit coated by laminin containing BDNF-CNPs, AuNPs-CNPs and filled with 2 × 10^6^ r-ADSCs suspended in fibrin matrix (PLGBCF) for each animal, fibrinogen (cryoprecipitate was used to prepare fibrinogen) concentration was used according to previous study [38].

Finally, 60 μl human fibrinogen containing 2 × 10^6^ cells with 40 μl thrombin (fresh frozen plasma (FFP) was used to prepare thrombin) was injected into the nerve conduit (FFP and cryoprecipitate were obtained from the Blood Bank of Isfahan Province, Isfahan, Iran).

The epineurium of the proximal and distal cut ends of the sciatic nerve were inserted 2 mm into the ends of the nerve conduit and sutured with a 10-0 nylon suture.

Then, the muscle layers and skin were re-approximated with 7-0 nylon sutures. Finally, the rats had free access to food and water, and light/dark cycle in the room consisted of 12/12 h (07:00–19:00) with artificial light.

### Sciatic function index

Functional recovery of the sciatic nerve was evaluated by the sciatic functional index (SFI) obtained from rat footprints on the white paper covered on the box floor. Three obvious footprints were selected from each rat, and finally three different parameters were measured: (i) heel to the third toe (print length, PL), (ii) the first toe to the fifth toe (toe spread, TS), and (iii) the second toe to the fourth toe (intermediate toe spread, ITS). The SFI was calculated by the following formula:

$${\text{SFI}}= - 38.3\left( {{\text{EPL}} - {\text{NPL}}} \right)/{\text{NPL}} + 109.5\left( {{\text{ETS}} - {\text{NTS}}} \right)/{\text{NTS}} + 13.3\left( {{\text{EIT}} - {\text{NIT}}} \right)/{\text{NIT}} - 8.8$$

E is the experimental sides, and N is the normal sides. Generally, a value of close to 0 indicates the normal function and a value of close to −100 indicates the higher impairment [39].

### Pinprick test

To determine of sensory recovery, the pinprick test was performed. The experimental limb of rats was pinched with standardized forceps from the toe to the heel. In the pinprick test limb withdraw response to painful stimulus was graded from 0 to 3. Pinprick test was evaluated from heel to toes and measured values include: 0 = no response, 1 = heel, 2 = dorsum of foot (mid-foot), and 3 = toes [40]^.^

### Electrophysiology

For each animal, electrophysiological testing was performed in a 12-week post-surgery period. After anesthesia, the electrophysiologic method was performed by placing stimulation electrode in the proximal end of the regenerated nerve and recording electrodes in the mid of gastrocnemius muscle. The compound muscle action potential (CMAP) has been used to estimate the numbers of repaired motor nerve fibers. The CMAP parameters were analyzed for each rat; for instance, the CMAP latency parameter was assessed from the stimulation site to the start of the response and measured in milliseconds.

In addition, the amplitude (AMP) of CMAPs was calculated by the potential difference between maximum negative and positive peaks of the CMAP signal in millivolts [41]. Electrophysiological analysis was calculated using the eProbe software. Finally, the CMAP parameters were compared among different groups.

### Muscle mass

After animals were euthanized by intraperitoneal (IP) injection with 100 mg/kg sodium pentobarbital solution (Sigma-Aldrich, St. Louis, MO, USA) [42], gastrocnemius muscles were removed from normal and injured sides and weighed while still wet by an electronic balance (A&D Weighing EK-3000I Portable Balance, 3000 g Capacity). The recovery rate and muscle atrophy were calculated by the wet weight of gastrocnemius muscle on the experimental side/the wet weight of gastrocnemius muscle on the normal contralateral side × 100% [43].

### Histological study

According to previous study, nerves and muscles from normal and surgical legs were removed and fixed [36].Then, paraffin blocks were cut into 5 μm thick sections. The transvers sections of gastrocnemius muscle were stained with Masson’s trichrome and examined under a light microscope. The mean diameter of the muscle fibers was measured using the Digimizer software.

The nerve paraffin blocks were cut into 5 μm thick cross sections with a microtome, stained with 0.125% toluidine blue solution, observed and evaluated by under a light microscope [44].

The G-ratio (the axon-to-fiber diameter ratio) is an important parameter to estimate degree of myelination [45]. The nine images at 400× magnifications were randomly selected to measure the mean number of myelinated axons. The five random fields were counted to calculate the mean numbers of myelinated nerve fibers. The average diameter of the myelin, axon and sciatic regenerated nerve fibers, and the mean number of myelinated axons were measured by the Digimizer software.

### Immunohistochemistry staining

In this study, S100 β as a specific marker for Schwann cells, myelin basic protein (MBP) as a myelin sheath marker, and neurofilaments-200 (NF200) as a growing axon marker were used. The 3 µm thick sections of nerves were prepared using a conventional microtome. Then, double immunohistochemical staining with anti-NF200 (Abcam, Cambridge, MA) and anti-S100β (Abcam, Cambridge, MA) or NF200 and MBP (Abcam, Cambridge, MA) were performed as described in our previous study [36]. Rabbit anti mouse FITC (Abcam, Cambridge, UK) and goat anti mouse Alexa Flour (Abcam, Cambridge, UK) were used as secondary antibodies.

Finally, all sections were incubated with 4,6-diamidino-2-phenylindole (DAPI) for cell nuclei staining, and immunohistochemistry results were examined under a fluorescent microscope (Olympus BX51, Japan). Then, the total area of the images was measured for the intensity of NF200, S100 and MBP staining using the Image-j software [46].

### Real-time reverse transcription polymerase chain reaction

The level of S100, MBP, GFAP and Nestin expression in the rat sciatic nerve tissue were assessed using real time RT-PCR. Total RNA was isolated using the Total RNA Prep Kit (BIOFACT). RNA was reverse transcribed using the BioFact™ 5X RT Pre-Mix cDNA Synthesis Kit (BIOFACT) according to the manufacturer’s protocol. The expression of target genes was evaluated using BioFact™ 2X Real-Time PCR Master Mix Kit (BIOFACT) through StepOne Plus™ quantitative Real Time PCR Detection System (Applied Biosystems). The level of β-actin was used as the control housekeeping gene. Table 2 lists the sequence of the used primers (metabion, Germany) [23].

**Table 2:** The list of primer sequences of *S100; Schwann cell marker, Nestin; Marker* of neuronal progenitor cells, *Gfap;* Glial fibrillary acidic protein, Mbp*;* Myelin basic protein, *β-actin;* As the control housekeeping gene for Real time RT-PCR analysis

Gene	Primer (forward (top) reverse (bottom))
*S100*	5′-ATAGCACCTCCGTTGGACAG-3′
	5′-TCGTTTGCACAGAGGACAAG-3′
*Nestin*	5′-CCGGGTCAAGACGCTAGAAGA-3′
	5′-CTCCAGCTCTTCCGCAAGGTTGT-3′
*Gfap*	5′-CTCCTATGCCTCCTCCGAGACGAT-3′
	5′-GCTCGCTGGCCCGAGTCTCTT-3′
*Mbp*	5′-CACAGAAGAGACCCTCACAGCGAC-3′
	5′-CCGCTAAAGAAGCGCCCGATGGA-3′
β-actin	5′-GTTGTCGACGACGAGCG-3′
	5′-GCACAGAGCCTCGCCTT-3′

### Statistical analysis

Statistical differences between the different groups were tested by the one-way ANOVA followed by post-hoc, tukey’s test using SPSS software version 22.0 (SPSS Inc., Chicago, IL, USA). Data were presented as mean ± standard error and *p*-values less than 0.05 were considered statistically significant.

## Data Availability

Data are available from the corresponding author on request.
